# Study on the influence of cementation sequence on the mechanical properties and microstructure of MICP-modified ili loess

**DOI:** 10.1371/journal.pone.0352646

**Published:** 2026-06-30

**Authors:** Guangming Shi, Ai Zhang, Yuan Xue, Dejun Yang, Changjiang Zhou

**Affiliations:** 1 School of Resources and Geosciences, China University of Mining and Technology, Xuzhou, China; 2 College of Geology and Mining Engineering, Xinjiang University, Urumqi, People’s Republic of China; 3 School of Environment and Spatial Informatics, China University of Mining and Technology, Xuzhou, China; Henan Polytechnic University, CHINA

## Abstract

The loess widely distributed in the Ili River Valley in north-western China is prone to soil erosion and geological hazards due to its high collapsibility, high soluble salt content and loose structure. This study investigates the effectiveness of Microbial-Induced Calcium Carbonate Precipitation (MICP) technology in improving the mechanical properties of Ili loess, focusing on a comparison of the macroscopic and microscopic properties under three different treatment sequences: bacterial suspension followed by cementation solution, cementation solution followed by bacterial suspension, and simultaneous application of both solutions. The results indicate that the treatment sequence significantly influences the MICP improvement effect, with the simultaneous application method performing best. Its unconfined compressive strength (115.83 kPa) increased by approximately 59.77% compared to untreated samples, and cohesion increased by approximately 55.06%, whilst the change in internal friction angle was minimal (<0.92%). Microscopic analysis indicated that the simultaneous application method formed a continuous, dense cementation network, effectively filling voids and bridging particles; in contrast, stepwise treatment tended to result in uneven distribution of the cementation material. It should be noted that this study constitutes a preliminary exploration under laboratory conditions; before the proposed method for optimising the cementation sequence can be applied to actual engineering projects, further research is required into durability (dry-wet/freeze-thaw cycles), scale effects and field validation. This study provides a potential environmentally friendly technical approach for the improvement of Ili loess.

## 1. Introduction

Loess, a Quaternary sediment characterised by its porous structure and weak cementation, is extensively distributed across regions such as the Loess Plateau and the Ili River Valley in Xinjiang, China [1–3]. Due to its pronounced engineering properties—including significant collapsibility and seismic collapse—this soil type is highly susceptible to structural failure when subjected to water or seismic loads. This leads to geological hazards such as loess landslides, posing severe threats to local socio-economic development and the safety of lives and property [4].

As a representative loess-bearing area in Xinjiang, the Ili River Valley serves not only as a vital transport hub connecting northern and southern Xinjiang and a significant tourist destination [5], but also hosts extensive critical infrastructure including water conservancy and transportation systems. However, its unique climatic conditions—characterised by seasonal aridity coupled with concentrated precipitation—render it one of Xinjiang's regions most frequently subjected to rainfall and freeze-thaw cycles [6]. Under the coupled effects of unique geotechnical conditions and extreme climate, geological hazards in the Ili River Valley have become increasingly prominent. For instance, the large-scale loess landslide that occurred in Zeketai, Xinyuan County, Ili in 2019 resulted in road destruction, river blockage, and the formation of a debris-dammed lake, posing a severe threat to the lives and property of surrounding residents [7]. Such disaster events underscore the urgent practical necessity of enhancing the engineering properties of loess in this region.

Currently, engineering improvements for loess primarily employ physical methods such as the subgrade method [8] and dynamic compaction [9], or chemical modification techniques using fly ash [10] and lignin [11]. While these conventional approaches can partially improve the soil's physical and mechanical properties, they generally suffer from high energy consumption during construction, potential disruption to the regional ecological environment, and the risk of secondary pollution [12]. Particularly in the ecologically fragile Ili River Valley, the insufficient environmental compatibility of traditional reinforcement techniques has become a key factor limiting their widespread application.

In recent years, microbial-induced calcium carbonate precipitation (MICP) technology has garnered significant attention as an emerging green geotechnical reinforcement method. This technique harnesses the metabolic activity of urease-producing microorganisms to generate calcium carbonate crystals within soil-rock bodies, thereby effectively cementing particles, filling pores, and improving soil-rock structure at the microscopic level. Compared to conventional physicochemical methods, MICP offers significant advantages including environmental friendliness, low energy consumption, and excellent compatibility [13–15]. In the broader field of seepage-mechanics coupling in rock and soil masses, researchers have recently explored the controlling role of medium heterogeneity on mechanical responses from multiple perspectives. For example, Cao et al. [16] investigated the seepage instability mechanism of karst sinkholes under mass-variation effects through numerical simulation, revealing the governing principles of particle migration and porosity evolution in the formation of water-conducting channels; Rong et al. [17] analysed the fracture propagation and mechanical degradation of granite under cyclic thermal loading and liquid nitrogen cryogenic shock, emphasising the decisive role of microstructural changes in macroscopic mechanical responses. The aforementioned studies provide important methodological references for understanding the mechanical behaviour of MICP-reinforced soils in complex environments.

MICP technology has demonstrated good adaptability across various complex soil types and environmental conditions. Tao et al. [18] identified 0.5 mol/L as the optimal binder concentration for palm fibre-reinforced MICP sand stabilization. Paul et al. [19] combined jute fibres with MICP to increase the strength of expansive soil by 186% and reduce its swelling strain by 85%. Sulaiman et al. [20] confirmed that polypropylene fibres significantly improve the post-peak ductility of MICP-stabilized river sand. Zhang et al. [21] used short coconut shell fibres (1–5 mm) to increase the compressive strength of stabilized marine unsaturated sandy soil by 98%. More recently, Paul and Islam [22] demonstrated the synergistic effect of bio-stimulated MICP combined with waste fibre reinforcement for stabilizing expansive subgrade soil, achieving notable improvements in strength and durability through the coupled action of calcite precipitation and fibre bridging. Collectively, these studies provide important methodological references for the ecological reinforcement of Ili Loess.

Existing research has confirmed MICP's effectiveness in improving sandy soils and general loess [23–29]. However, systematic studies on the highly saline, strongly collapsible loess found in the Ili region remain scarce. While existing parameter optimisation studies on MICP-treated loess [30,31] have primarily focused on factors such as calcium source type, binder concentration, and freeze-thaw durability, the influence of the treatment sequence—i.e., the order of bacterial suspension and cementation solution application—on consolidation effectiveness has yet to be systematically investigated. More critically, MICP treatment efficacy is highly dependent on the reaction process. The application sequence directly influences microbial distribution within the soil and their spatial relationship with reactants, constituting a key determinant of consolidation uniformity and efficiency. This factor, however, remains under-explored in existing literature.

Consequently, this study focuses on loess from Xinjiang's Ili region. Through systematic laboratory experiments, it investigates the impact of three distinct treatment sequences (bacterial suspension followed by cementation solution, cementation solution followed by bacterial suspension, and simultaneous application of both solutions) on MICP improvement efficacy. The study employs a comprehensive methodology incorporating unconfined compressive strength tests, triaxial shear tests, and scanning electron microscopy (SEM) micrographs to elucidate the macro- and micro-mechanisms through which the treatment sequence influences consolidation outcomes. This research aims to provide critical process parameters and theoretical foundations for the potential application of MICP technology in engineering consolidation of loess in the Ili River Valley, thereby contributing to the development of sustainable solutions for geological hazard prevention and ecological slope protection in this region.

## 2. Experimental Materials and methods

### 2.1. Experimental materials

#### 2.1.1. Test soil samples.

The loess used in the experiments was collected from a typical loess slope in Nilek County, Ili Prefecture, Xinjiang. As the site is not located within a protected area and no endangered or protected species were involved, no special permits were required for access or sampling under local or national regulations. Sampling was conducted at a depth of 1.5–2.0 metres below ground level to avoid the influence of surface vegetation and weathering. The undisturbed soil samples obtained were sealed for preservation and transported to the laboratory. The soil samples exhibited an earthy yellow colour and a relatively compact structure.

The particle size distribution was determined using sieve analysis. Results indicated that the Ili loess consisted predominantly of silt particles (0.075–0.005 mm), accounting for 77.2% of the composition; followed by sand particles (>0.075 mm) at 17.2%; and clay particles (<0.005 mm) at 5.6%. Standard compaction tests determined the optimum moisture content of the soil sample to be 18.77% and the maximum dry density to be 1.87 g/cm^3^.

Basic physical properties of the soil were determined according to the Standard Test Methods for Soil (GB/T 50123−2019) [32], with results presented in Table 1.

**Table 1:** Soil physical properties.

Natural moisture content(w%)	Natural density (g/cm^3^)	Dry density (g/cm^3^)	Liquid limit(%)	Plastic limit(%)	Optimum moisture content(w%)	Maximum dry density (g/cm^3^)	Soluble salt content (mg/kg)
16.49	16.0	1.38	25.87	17.08	18.77	1.87	1307.5

#### 2.1.2. Microbial cultivation and concentration/activity assays.

Microbial-induced calcium carbonate precipitation (MICP) constitutes a biologically induced mineralisation process, with microbial urease activity serving as its pivotal influencing factor. To ensure the efficiency and controllability of the mineralisation process, it is essential to obtain bacterial cultures exhibiting high concentration and stable, elevated activity [33].

##### 2.1.2.1 Test strain

*Sporosarcina pasteurii* (also known as *Bacillus pasteurii*) has been widely selected as an engineered strain for MICP technology due to its prominent urease activity and adaptability in complex environments. The strain employed in this study, *Sporosarcina pasteurii* (ATCC 11859), belongs to the order Bacillales, family *Sporosarcinacea*e, genus *Sporosarcina*, and was procured from the China General Microbiological Culture Collection Centre (CGMCC). This bacterium is an aerobic Gram-positive organism exhibiting high tolerance to ammonium ions, the hydrolysis product of urea [34,35], rendering it suitable for soil improvement applications. Prior to experimentation, the freeze-dried culture was revived through subculture and activated for subsequent use.

##### 2.1.2.2 Preparation of culture medium

Microbial growth and enzyme production depend on the nutrients provided by the culture medium. In this experiment, CM0907 urea medium was used; the specific composition is shown in Table 2. To prevent the decomposition of urea caused by high temperatures, a stepwise sterilisation method was employed: after dissolving the base components of the medium (excluding urea), the mixture was sterilised at 121°C for 20 minutes in an autoclave. Concurrently, a urea solution was prepared separately and filtered through a 0.22 μm filter membrane to remove bacteria. Once the base medium had cooled to room temperature, the urea solution was added under sterile conditions in a clean bench, mixed thoroughly, and set aside. All operations were carried out in a clean bench to ensure sterility.

**Table 2:** Composition of culture media.

CM0907 Medium		
Peptone	5.00	g
Beef extract	3.00	g
Urea	20.00	g
Distilled water	1.00	L
pH	7	

1 mol/L sodium hydroxide solution is used to adjust the medium to pH 7.0.

##### 2.1.2.3 Strain activation and expansion culture

The strain activation procedure is as follows: Add 0.3 mL of liquid medium to the freeze-dried bacterial powder ampoule. Gently shake to dissolve, then transfer to a test tube containing 4.5 mL of sterile liquid medium. Mix thoroughly to obtain the stock solution. Dilute the stock solution to a concentration of 10^−9^ using a tenfold serial dilution method. Inoculate the diluted culture onto solid medium using the streak plate method. Incubate at 30°C in a constant-temperature incubator for 24 hours. Select morphologically sound single colonies and inoculate them into liquid medium. Place in a constant-temperature shaking incubator for expansion culture at 30°C and 180 r/min. This culture is used for subsequent growth curve analysis and enzyme activity assays.

##### 2.1.2.4 Bacterial culture concentration and urease activity assay

To determine the optimal incubation time for bacterial cultures used in MICP tests, the dynamic changes in bacterial culture concentration (OD₆₀₀) and urease activity were systematically measured.

Bacterial Culture Concentration Measurement: Using a UV spectrophotometer, the absorbance of the bacterial culture (OD₆₀₀) was measured at a wavelength of 600 nm. Samples were taken every two hours for continuous monitoring over 36 hours, with calculations performed according to Equation (1) [36]. The instrument was preheated for 20 minutes prior to measurement, and uninoculated medium served as the blank control.


$$\begin{array}{c} Y=\text{8}.\text{5}\text{9}\times {\text{1}\text{0}}^{\text{7}}\times Z^{\text{1}.\text{3}\text{6}\text{2}\text{7}} \end{array}$$ (1)

Y: Cell concentration (cells/mL);

Z: Represents the OD600 value, i.e., the absorbance.

Urease activity assay: Employing the conductivity method. Add 3 mL of bacterial suspension to 27 mL of 1.11 mol/L urea solution. Using a conductivity meter, record the change in solution conductivity ΔEc over 5 minutes. Urease activity (U, units mmol/L/min) is calculated according to Equation (2) [37]:


$$\begin{array}{c} U=\frac{\Delta Ec}{\Delta t}\times \text{10}\times \text{11}.\text{11} \end{array}$$ (2)

*U:* Urease activity (expressed as urea hydrolysis rate), (mmol/L/min);

$\frac{\Delta Ec}{\Delta t}$: Average rate of change in reaction solution conductivity over time (mS/cm/min);

Coefficient 10: Medium diluted tenfold; multiply by 10 to obtain enzyme activity in the original sample;

Coefficient 11.11: An increase of 1 mS/cm in conductivity corresponds to 11.11 mM urea hydrolysis.

**Fig 1:**
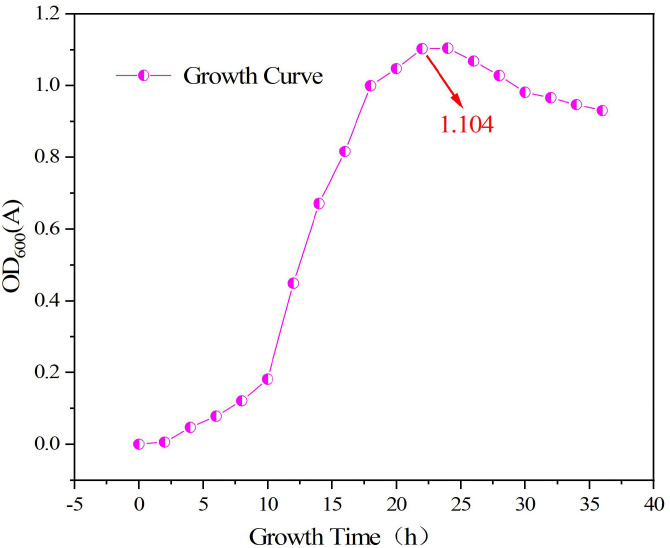
Microbial growth curve diagram.

**Fig 2:**
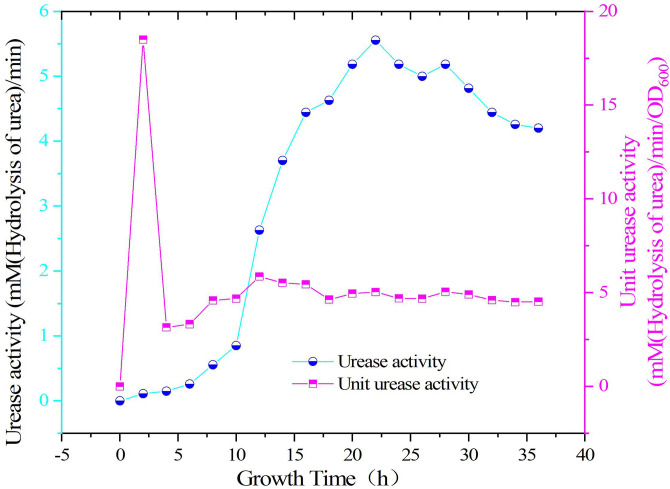
Enzyme activity curve.

Changes in bacterial culture concentration and urease activity over time are shown in Figs 1 and 2. The optical density at 600 nm (OD₆₀₀) of the bacterial culture peaked at approximately 1.11 A after 20–24 hours of incubation, corresponding to the stationary phase of bacterial growth. The trend in urease activity largely synchronised with bacterial growth, reaching a maximum value of approximately 5.56 mS·cm^−1^·min^−1^ around 20 hours.. Enzyme activity per unit bacterial mass rose rapidly during the early culture phase before gradually declining and stabilising.

Taking into account microbial growth, total enzyme activity, and changes in specific enzyme activity, this study selected bacterial cultures incubated for 20–24 hours for subsequent MICP experiments. During this period, bacterial concentration is high and enzyme activity is stable, facilitating efficient and controllable calcium carbonate precipitation.

#### 2.1.3. Binding solution.

The cementation solution provides a urea nitrogen source for bacterial growth and a calcium source for the MICP reaction process [33]. In studies on the modification of the MICP process, calcium chloride, calcium acetate and calcium nitrate are commonly used as calcium sources; among these, calcium chloride is widely employed due to its significant advantages in promoting bacterial growth, maintaining urease activity and increasing the yield of calcium carbonate. The binder solution used in this study was prepared from 0.5 mol/L urea and 0.5 mol/L calcium chloride (CaCl_2_) (molar ratio 1:1), with the pH adjusted to 8.0 using 1 M NaOH solution.

### 2.2. Sample preparation and MICP treatment

In this experiment, the soil samples were treated using the spray-mixing method. To prepare soil samples with a moisture content of 18.77% (the optimum moisture content), an equal mass of bacterial suspension (OD_600_ = 1.1) was first mixed thoroughly with a cementation solution (0.5 M urea + 0.5 M CaCl_2_). This mixture was then used in place of distilled water to mix with the loess, thereby bringing the soil samples to the target moisture content [38]. To ensure uniform mixing, the mixture was stirred manually. During the stirring process, the mixture was sprayed evenly onto the soil sample in batches, with stirring carried out simultaneously. The total stirring time was approximately 30 minutes, until the colour and moisture content of the soil sample were completely uniform.

**The key variable in this experiment is the cementation sequence. Three groups were established, with the total reaction time—defined as the duration during which the bacterial suspension and cementation solution coexist in the soil matrix—controlled at 6 hours for all groups:**

**Group A (bacteria first): Bacterial suspension was sprayed and mixed with soil, followed by a 30-minute pre-treatment period for bacterial adsorption (during which no cementation solution was present). Then, the cementation solution was added and mixed, and the reaction proceeded for 6 hours before sample preparation.**

**Group B (cementation solution first): Cementation solution was sprayed and mixed with soil, followed by a 30-minute pre-treatment period. Then, the bacterial suspension was added and mixed, and the reaction proceeded for 6 hours before sample preparation.**

**Group C (simultaneous): Bacterial suspension and cementation solution were pre-mixed, then sprayed onto the soil and mixed, followed by a 6-hour reaction period before sample preparation.**

**Fig 3:**
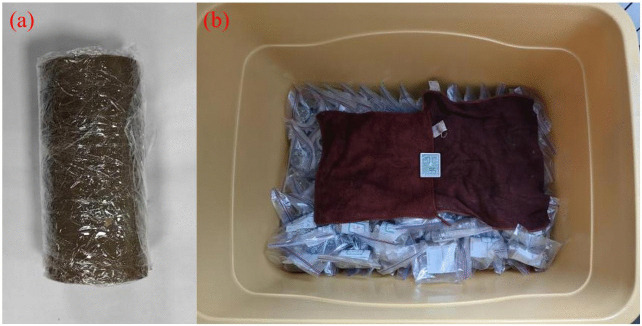
Photographs of specimens and curing process. (a: Photograph of the specimen; b: Photograph of the specimen during curing).

The prepared soil samples were divided into three layers and compacted sequentially into a Φ39.1 mm × 80 mm mould, achieving 90% compaction. Samples were then wrapped in cling film, sealed in plastic bags, and placed in storage boxes for 14 days of curing (Fig 3). During curing, temperature was maintained at approximately 30°C and humidity at around 95%.

Specimens for each treatment group (Groups A, B, C and the untreated control group) were prepared from the same batch, using homogenised soil samples and bacterial/cementation solutions from the same batch. The allocation of specimens for each test item is shown in Table 3. Three parallel specimens were prepared for both the UCS and triaxial tests, with results expressed as mean ± standard deviation. For SEM observation and pore image analysis, three representative locations were selected from the post-failure specimens of each treatment group for characterisation.

**Table 3:** Allocation of test specimens for each test item.

Test item	Treatment group	Number of replicates	Total number of samples	Remarks
Unconfined compressive strength test (UCS)	Group A / Group B / Group C / Control group	3	12	
Triaxial shear test (confinement pressure: 100, 200, 300 kPa)	Group A / Group B / Group C / Control group	3	36	
Scanning electron microscopy (SEM)	Group A / Group B / Group C / Control group	3	12	
Pore image analysis	Group A / Group B / Group C / Control group	3	12	Shared samples with SEM
Total			60	

### 2.3. Testing and characterisation methods

#### 2.3.1. Unconfined compressive strength test.

Following MICP treatment and curing, measurements were conducted using an STK.WCX-II unconfined compressive strength testing apparatus at a constant rate of 0.5 mm/min. The peak strength recorded was noted as the unco.nfined compressive strength (UCS).

#### 2.3.2 Triaxial shear test.

(1) Basis for the selection of test methods
According to the three-factor classification of sediments, the Ili loess is classified as sub-clay [39]; it has relatively small soil particles and a low permeability coefficient. Following MICP treatment, the precipitation of calcium carbonate further fills the intergranular pores, resulting in a permeability coefficient that is an order of magnitude lower than that of untreated loess [40]. Under these circumstances, if the undrained consolidation (CU) or drained consolidation (CD) test methods are employed, the consolidation phase not only requires an extremely long drainage time but also makes it difficult to accurately determine whether the specimen has reached theoretical full consolidation. More critically, slope instability in actual engineering projects typically occurs under instantaneous conditions where ‘there is no time for drainage’, rather than under conditions of ‘loading after full drainage’. Therefore, to better reflect the rapid loading scenarios that may occur in actual engineering projects (such as instantaneous strength changes triggered by rainfall infiltration), this study adopted the undrained unconsolidated (UU) shear test scheme.
(2) Condition of test specimens
The moisture content of the test specimens was maintained at the optimum value of 18.77%; based on calculations of the specific gravity and dry density of the soil, the corresponding degree of saturation was approximately 85%.
(3) Test Equipment and Conditions
A ‘TKA-TTS-1U’ unsaturated soil stress-strain controlled triaxial tester was used to conduct tests under three different confining pressures: 100 kPa, 200 kPa and 300 kPa. The drainage valve was closed during the shearing process to ensure undrained conditions. The axial loading rate was set at 0.5 mm/min, which is sufficiently rapid to effectively maintain undrained conditions in low-permeability soils. The test was terminated when the axial strain reached 15% or when the specimen showed signs of significant failure.
(4) Failure criteria
If the stress-strain curve exhibits a distinct peak, this peak value shall be taken; if no distinct peak is present, the deviatoric stress value corresponding to 15% of the axial strain shall be taken.
(5) Calculation of Shear Strength Parameters
Based on the total stress method, calculate the cohesion (c) and angle of internal friction (φ) using the Mohr–Coulomb failure criterion.
First, calculate the centre (p) and radius (r) of the Mohr circle for each specimen within the same treatment group under different confining pressures using the following equation:

$$\begin{array}{c} \text{p}=(\sigma_{\text{1}}+\sigma_{\text{3}})\;/\;\text{2} \end{array}$$ (3)

$$\begin{array}{c} \text{r}=(\sigma_{\text{1}}-\sigma_{\text{3}})\;/\;\text{2} \end{array}$$ (4)

Where: σ_1_ is the principal stress; σ_3_ is the secondary principal stress

Next, plot the Mohr’s circles for different confining pressures, with p on the x-axis and r on the y-axis. Fit a common tangent through each Mohr’s circle to obtain the Mohr–Coulomb failure envelope. The y-intercept of this envelope is the total stress cohesion c, and the angle corresponding to its slope is the total stress angle of internal friction φ.

It should be noted that, as the UU test does not measure pore water pressure, all calculations are based on total stress; the strength parameters obtained correspond to the total stress shear strength under undrained conditions.

#### 2.3.3. Microscopic structure observation.

To elucidate the microscopic mechanisms underlying changes in macroscopic mechanical properties, observations were conducted using scanning electron microscopy (SEM). Specimens were thoroughly dried and cut into squares of approximately 1 cm × 1 cm. These were subjected to vacuum treatment and gold sputtering for 90 seconds before being placed in the SEM for imaging, thereby acquiring SEM micrographs. The arrangement of soil particles and pore morphology were examined, with particular focus on the morphology and size of calcium carbonate crystals, as well as their adhesion and bridging behaviour between soil particles.

## 3. Results and analysis

### 3.1. Unconfined compressive strength (UCS) test

**Fig 4:**
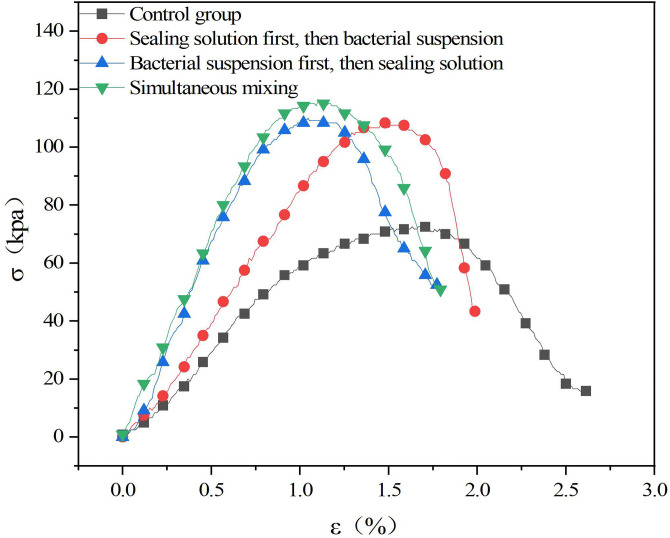
Stress-strain curve diagram.

To evaluate the impact of different application sequences on the reinforcement effect of Microbial-Induced Calcium Carbonate Precipitation (MICP), unconfined compressive strength tests were conducted on four specimen groups: (1) untreated control group; (2) bacterial suspension first followed by cementation solution; (3) cementation solution first followed by bacterial suspension; and (4) simultaneous application of both solutions. Stress-strain curves were obtained, as illustrated in Fig 4.

**Fig 5:**
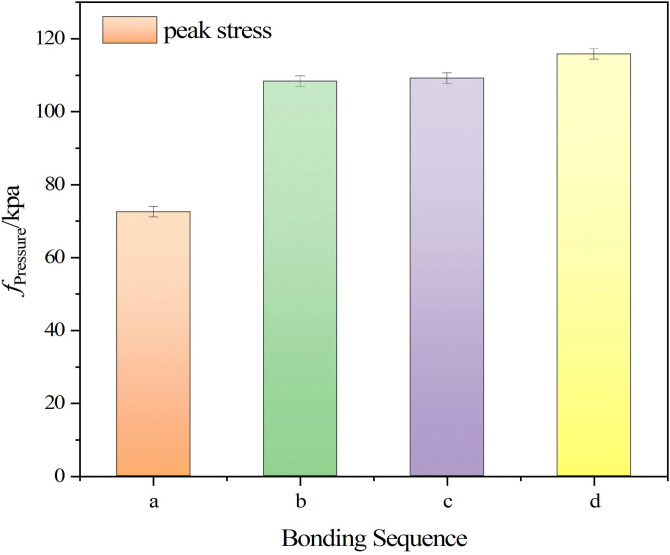
Bar chart of unconfined peak compressive strength. (a = Control group; b = Binder solution first, then bacterial suspension; c = Bacterial suspension first, then binder solution; d = Simultaneous mixing).

A comprehensive analysis combining Figs 4 and 5 indicates that the treatment sequence has a significant effect on the strength of the specimens. The unconfined compressive strength of the control group was 72.50 ± 1.44 kPa (n = 3).

Group C (simultaneous application) exhibited the highest peak mechanical strength. The stress in this group increased rapidly during the initial loading phase and reached a peak strength of 115.83 ± 0.83 kPa at an axial strain of approximately 1.1%, representing an increase of approximately 59.77% compared to the control group. The stress-strain curve for this group remained at the highest level throughout the entire test, indicating that the simultaneous mixing method maximally promoted the thorough mixing and uniform precipitation of the cementation material, resulting in a well-integrated cemented structure.

Group A (bacteria first) showed relatively rapid strength development, with a peak strength of 110.00 ± 1.44 kPa, representing an increase of approximately 51.72% compared to the control group. After reaching its peak at a strain of approximately 1.0%, the strength began to decline, exhibiting certain brittle characteristics.

Group B (cementation solution first) showed slower strength development, entering a rapid growth phase only in the later stages of strain. It ultimately reached a peak strength of 108.33 ± 1.44 kPa at a strain of approximately 1.67%, representing an increase of approximately 49.42% compared to the control group.

A one-way analysis of variance revealed a highly significant difference in unconfined compressive strength among the treatment groups (F = 811.95, P < 0.001). Levene's test indicated that the variances were homogeneous across all groups (P = 0.787 > 0.05). Further multiple comparisons using the LSD method revealed that the strength of all MICP treatment groups was extremely significantly higher than that of the control group (P < 0.001); the strength of Group C was significantly higher than that of Group A (P < 0.001) and Group B (P < 0.001); and the strength of Group A was significantly higher than that of Group B (P = 0.048). The reinforcement effectiveness of the three treatment sequences was ranked as follows: Group C > Group A > Group B, which was fully consistent with the results of the statistical tests.

In terms of failure behaviour, the control group and Group B reached their peak at higher strains (>1.65%), retaining some of the plastic characteristics of the Ili loess. Conversely, Group A and Group C reached their peak at lower strains (1.00–1.10%), exhibiting stronger brittle characteristics; this represents a typical shift in mechanical behaviour following effective cementation of the soil by calcium carbonate.

### 3.2. Triaxial shear strength characteristics

#### 3.2.1. Stress-strain curves.

**Fig 6:**
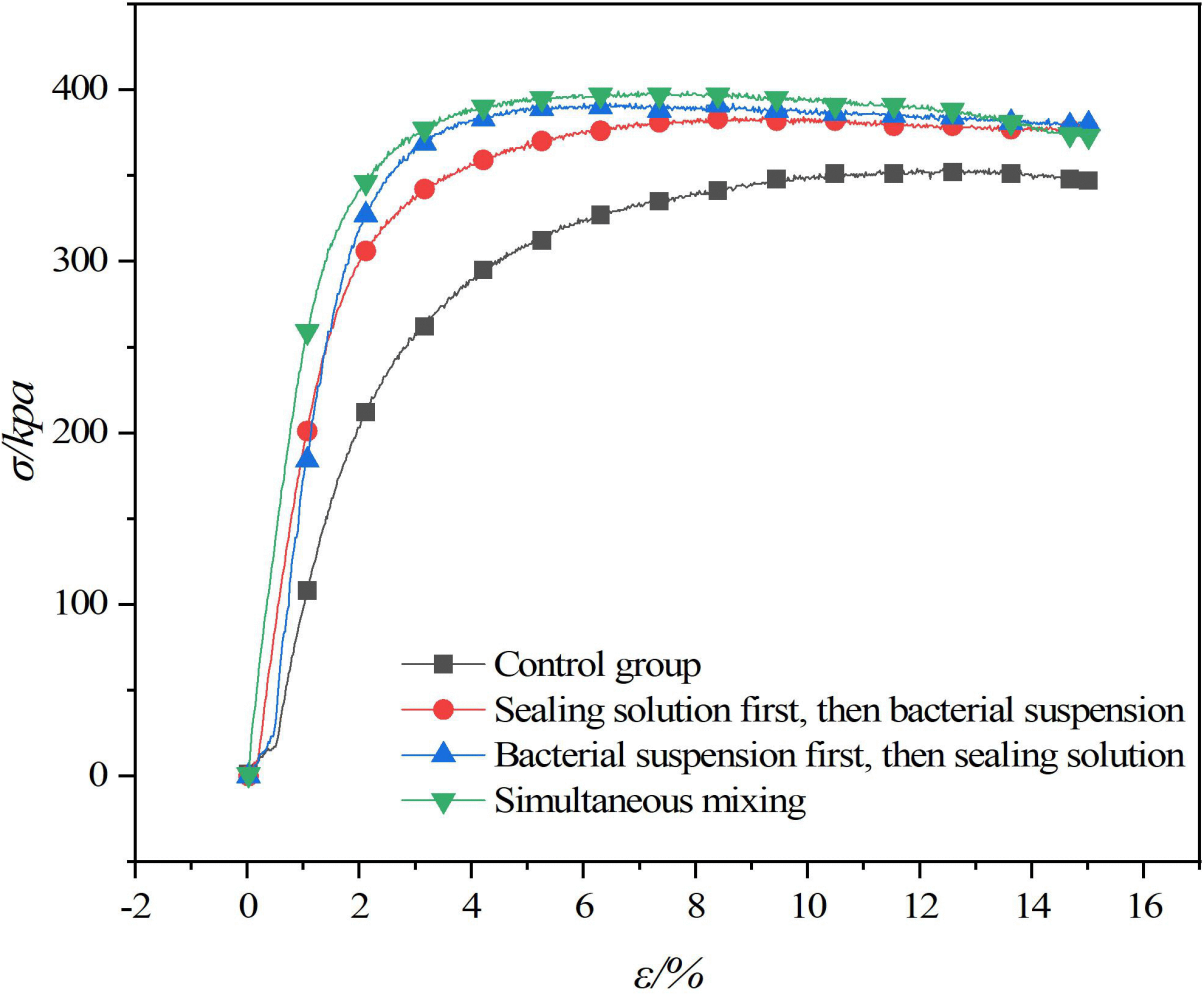
100kpa axial strain-shear stress curves.

**Fig 7:**
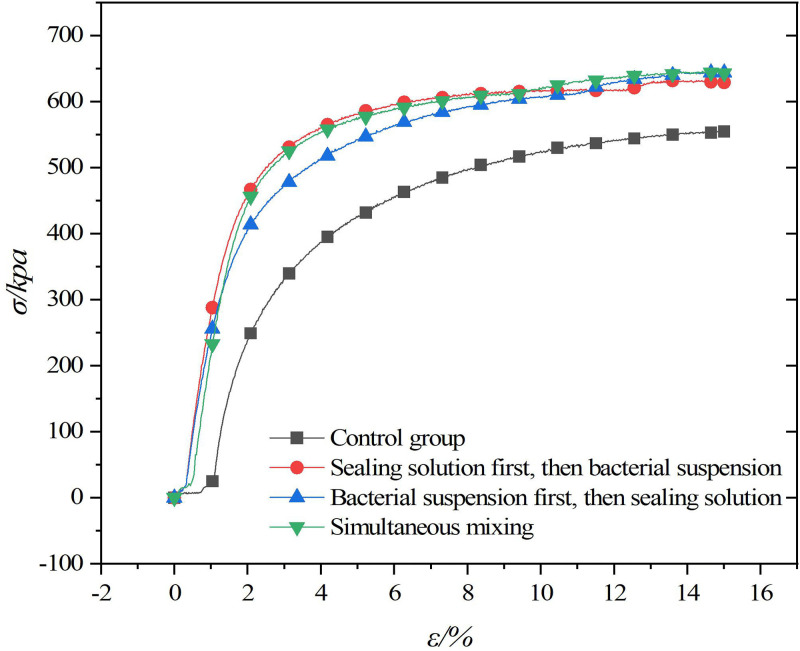
200kpa axial strain-shear stress curves.

**Fig 8:**
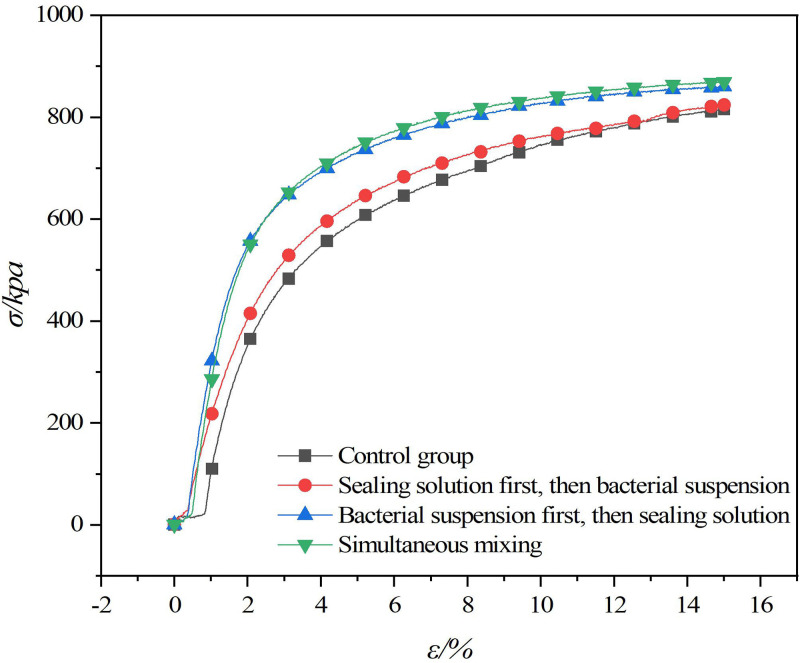
300kpa axial strain-shear stress curves.

The triaxial shear strength of specimens is a key indicator for assessing their stability and load-bearing capacity, providing a crucial basis for engineering applications. In this study, the axial strain-shear stress relationship curves for untreated specimens and specimens subjected to different treatment sequences are presented at three distinct confining pressures (100 kPa, 200 kPa, and 300 kPa), as shown in Figs 6–8.

**Fig 9:**
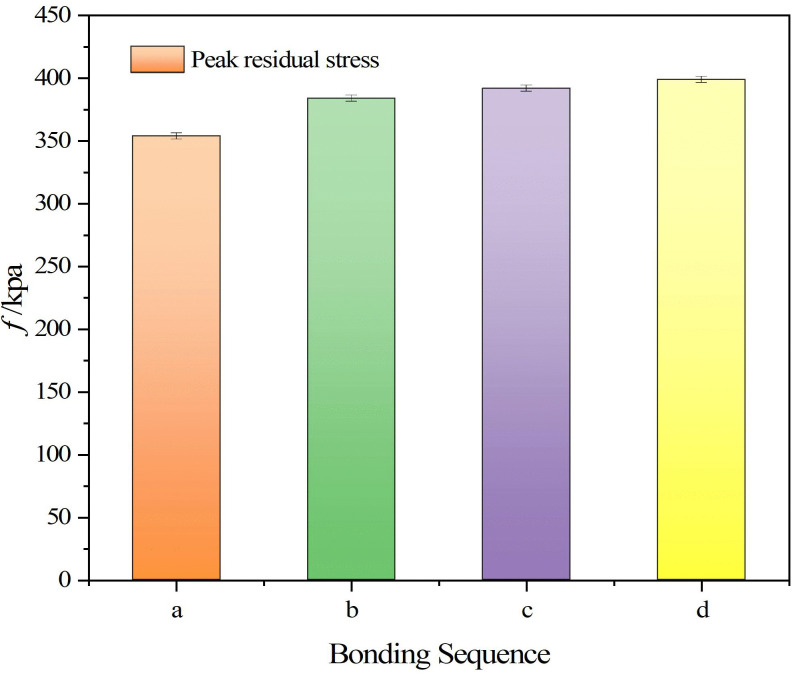
100kpa peak stress diagrams for different cementation sequences under varying confining pressures. (a = Control group; b = Binder solution first, then bacterial suspension; c = Bacterial suspension first, then binder solution; d = Simultaneous mixing).

Under a confining pressure of 100 kPa, the curves for the different treatment groups showed a clear distinction in the early stages of strain (<5%) and gradually converged as strain increased (Fig 6), exhibiting a certain degree of strain softening. As shown in Fig 9, the peak strength of the control group was 354 ± 2.45 kPa (n = 3). Following treatment with different sequences, the peak strengths of Group B (cementation first), Group A (bacteria first) and Group C (simultaneous application) increased to 384 ± 2.45 kPa, 392 ± 2.45 kPa and 399 ± 2.45 kPa, respectively, representing increases of 8.78%, 11.05% and 13.03% compared with the control group. One-way ANOVA revealed extremely significant differences in peak stress between the treatment groups (F = 420.55, P < 0.001). Levene's test indicated homogeneity of variances across the groups (P = 0.210 > 0.05). Further multiple comparisons using the LSD method revealed that the peak deviator stress in all MICP treatment groups was significantly higher than that in the control group (P < 0.001); Group C was significantly higher than Group A (P < 0.001) and Group B (P < 0.001); and Group A was significantly higher than Group B (P < 0.001). The reinforcement effects of the three treatment sequences were ranked as follows: Group C > Group A > Group B.

**Fig 10:**
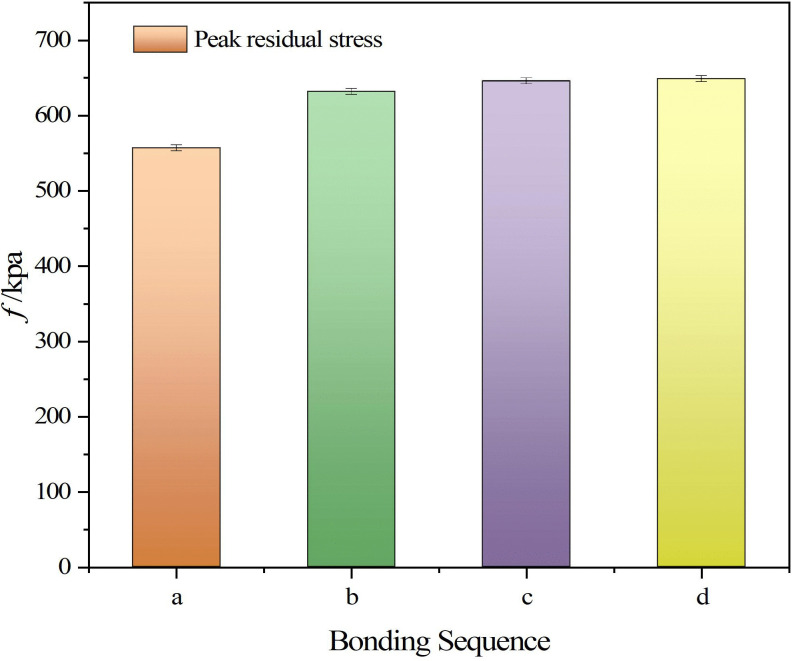
200kpa peak stress diagrams for different cementation sequences under varying confining pressures. (a = Control group; b = Binder solution first, then bacterial suspension; c = Bacterial suspension first, then binder solution; d = Simultaneous mixing).

Under a confining pressure of 200 kPa, the stress–strain curves of specimens subjected to different treatment sequences exhibited more complex, phased characteristics (Fig 7). As shown in Fig 10, the peak strength of the control group was 557 ± 4.06 kPa (n = 3). Following treatment with different sequences, the peak strengths of Group B, Group A and Group C increased to 632 ± 4.06 kPa, 646 ± 4.06 kPa and 649 ± 4.06 kPa, respectively, representing increases of 13.46%, 15.62% and 16.16% compared to the control group. One-way ANOVA revealed highly significant differences in peak stress between the treatment groups (F = 703.91, P < 0.001). Levene's test indicated that the variances were homogeneous across all groups (P = 0.062 > 0.05). Further multiple comparisons using the LSD method revealed that the peak shear stress in all MICP treatment groups was significantly higher than that in the control group (P < 0.001); Group C was significantly higher than Group B (P < 0.001); Group A was significantly higher than Group B (P = 0.001); whilst the difference between Group C and Group A did not reach statistical significance (P = 0.126). This may be partly due to the confining pressure masking some differences in the cemented structure, whilst also being limited by the statistical power of the small sample size (n = 3); further validation could be achieved in subsequent studies by increasing the sample size.

**Fig 11:**
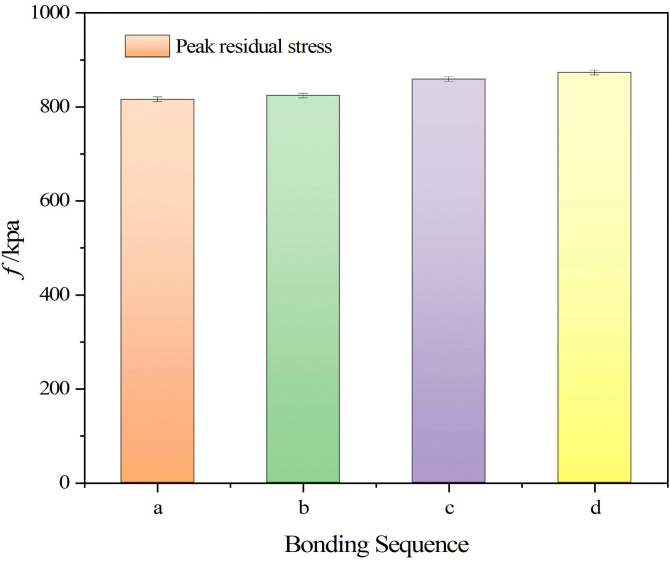
300kpa Peak stress diagrams for different cementation sequences under varying confining pressures. (a = Control group; b = Binder solution first, then bacterial suspension; c = Bacterial suspension first, then binder solution; d = Simultaneous mixing).

Under a confining pressure of 300 kPa, the strengthening effect of the confining pressure itself was dominant, whilst the contribution of the improved treatment sequence was relatively reduced (Fig 8). As shown in Fig 11, the peak strength of the control group was 816 ± 5.02 kPa (n = 3). Following treatment with different sequences, the peak strengths of Group B, Group A and Group C increased to 824 ± 5.02 kPa, 859 ± 5.02 kPa and 873 ± 5.02 kPa, respectively, representing increases of 0.98%, 5.27% and 6.99% compared with the control group. One-way ANOVA revealed extremely significant differences in peak shear stress among the treatment groups (F = 168.59, P < 0.001). Levene's test indicated homogeneity of variances across the groups (P = 0.109 > 0.05). Further multiple comparisons using the LSD method revealed that the peak deviatoric stress in all MICP treatment groups was significantly higher than that in the control group (Group B: P = 0.042; Groups A and C: P < 0.001); Group C was significantly higher than Group A (P = 0.006) and Group B (P < 0.001); and Group A was significantly higher than Group B (P < 0.001).

Overall, in the samples treated by simultaneous mixing, the simultaneous addition of the bacterial suspension and the cementation solution ensured that the reactants were mixed instantly, thoroughly and uniformly within the soil matrix. This facilitated the rapid and homogeneous nucleation and precipitation of calcium carbonate within the pore spaces, resulting in a well-distributed cementation network; in contrast, for samples treated with bacterial suspension followed by cementation solution, bacteria colonise the soil particle surfaces first, providing a large number of active enzymatic reaction sites for the subsequently added cementation solution. This promotes the more direct and effective precipitation of calcium carbonate at particle contact points, resulting in high cementation efficiency. Finally, the treatment method involving the application of the cementation solution followed by the bacterial suspension may result in partial loss of the cementation solution or suboptimal reaction sites due to a lack of sufficient bacterial catalysis. Consequently, the subsequently added bacterial suspension is unable to effectively utilise the pre-existing environment, leading to a decrease in the nucleation rate and spatial distribution uniformity of calcium carbonate precipitation, thereby limiting the overall cementation strength [41]. Furthermore, an increase in confining pressure enhances the skeletal strength of all specimens (including the control group) by increasing inter-particle friction. It also provides stronger lateral confinement for the calcium carbonate crystals generated by microbial activity, allowing the cementation process to function more robustly. This is reflected in the fact that the strength of all specimens increases with rising confining pressure.

#### 3.2.2. Evolutionary patterns of shear strength parameters.

**Fig 12:**
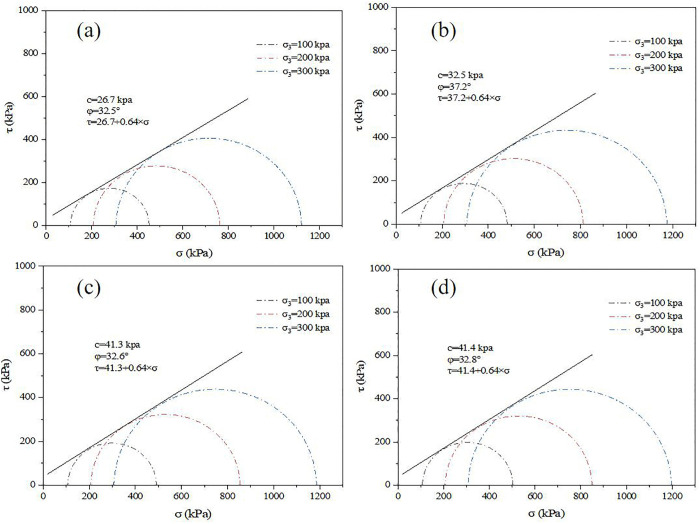
Mohr-Coulomb Envelope. (a = Control group; b = Cementation solution first, then bacterial suspension; c = Bacterial suspension first, then cementation solution; d = Simultaneous application).

**Fig 13:**
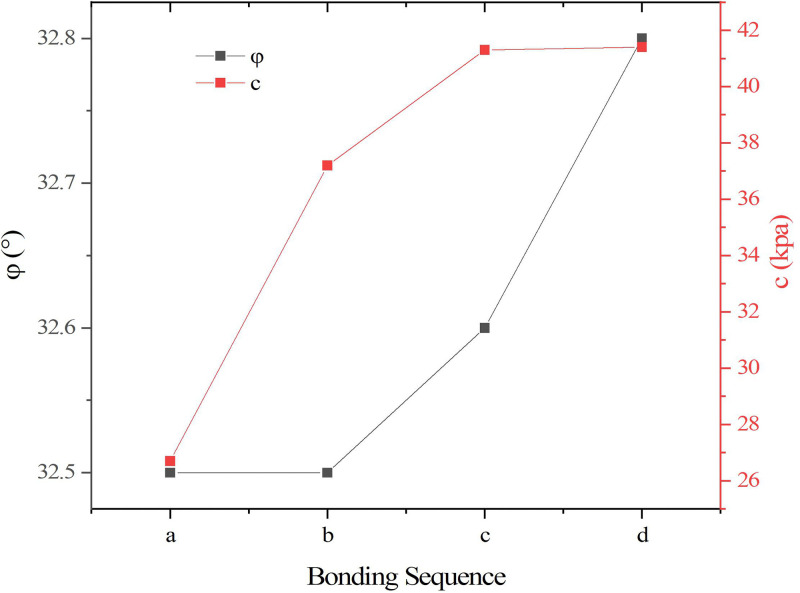
Relationship diagram between φ and bonding sequence. (a = Control group; b = Cementation solution first, then bacterial suspension; c = Bacterial suspension first, then cementation solution; d = Simultaneous application).

Based on the stress-strain curves obtained from the triaxial tests, the Mohr-Coulomb envelope was plotted (Fig 12). The cohesion (c) and internal friction angle (φ) for the specimens under different treatment sequences were determined, as shown in Table 4. The relationship between cohesion, internal friction angle, and treatment sequence is presented in Fig 13.

**Table 4:** Cohesion (c) and Internal friction angle (φ).

cementation sequence	Control group	Cementation solution first, then bacterial suspension	Bacterial suspension first, then sealing solution	Simultaneous application
Φ(°)	32.5	32.5	32.6	32.8
C(kPa)	26.7	37.2	41.3	41.4

Analysis of the cohesion (c) and angle of internal friction (φ) in Table 4 indicates that the soil improvement effect of the microbial-induced calcium carbonate precipitation (MICP) technique on the Ili loess is primarily reflected in an increase in cohesion. Compared with the untreated control group (c = 26.7 kPa), the cohesion of samples treated with the three different treatment sequences increased by 39.33% (cementation solution first followed by bacterial suspension; c = 37.2 kPa), 54.68% (bacterial suspension first followed by cementation solution; c = 41.3 kPa) and 55.06% (simultaneous application; c = 41.4 kPa), respectively, with the best improvement observed in the simultaneous application treatment. This indicates that the calcium carbonate crystals generated by MICP effectively enhanced the cementation between soil particles. At the same time, the change in the angle of internal friction within each treatment group was minimal (φ = 32.5°–32.8°), with a maximum increase of only 0.92%, suggesting that this technology primarily improves particle bonding through biomineralisation, whilst leaving the original particle characteristics and friction properties of the soil largely unchanged.

### 3.3. Microstructural analysis

To elucidate the mechanism by which the cementation sequence influences the improvement effectiveness of microbial-induced calcium carbonate precipitation (MICP), systematic microstructural observations and analyses were conducted on specimens treated with three distinct cementation sequences using scanning electron microscopy (SEM).

#### 3.3.1. Microstructural analysis of different setting sequences.

**Fig 14:**
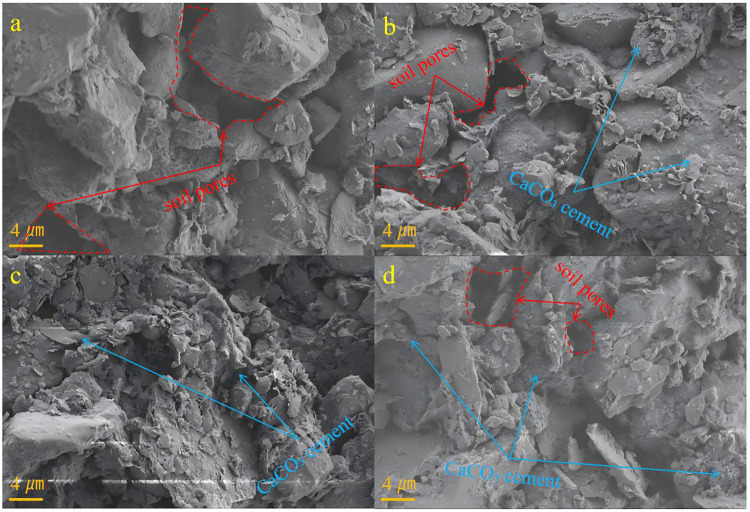
Microscopic images of different cementation sequences. (a = control group; b = cementation solution followed by bacterial suspension; c = bacterial suspension followed by cementation solution; d = simultaneous application).

SEM observations reveal significant differences in the microstructure and distribution of calcium carbonate precipitates among specimens subjected to different cementation sequences, as illustrated in Fig 14.

As shown in Fig 14a, the microstructure of the control sample is relatively loose, with a large number of voids between soil particles. However, these are predominantly large voids with poor connectivity [42]. Fig 14b–14d show the microstructural images of the modified samples, respectively, demonstrating a significant reduction in pore area within the soil following microbial modification. The calcium carbonate crystals induced by microorganisms are predominantly block-like or granular in shape; they agglomerate to form aggregates, filling the intergranular voids and cementing adjacent soil particles or adhering to the surfaces of soil particles [43].

Specifically, in Fig 14b (sample treated with cementation solution followed by bacterial suspension), the amount of calcium carbonate formed is relatively small and is mainly distributed on the surface of soil particles. Although the number of pores is significantly reduced compared to the control group, a considerable number of pores remain.

In Fig 14c (sample treated with bacterial suspension followed by cementation solution), the number of pores is low, and the calcium carbonate formed consists mainly of dense granular aggregates concentrated between particles to act as a cementing agent, resulting in higher mechanical strength.

Fig 14d (simultaneous application treatment sample) shows the fewest pores with the smallest pore sizes. Large block-like calcium carbonate crystals cement the particles, and the particle surfaces are also covered by a thin layer of calcium carbonate; hence, it exhibits the highest mechanical strength.

A comparison of the mechanical properties and SEM images of the samples treated with bacterial suspension followed by cementation solution and those treated by simultaneous application reveals that the improvement in the soil's mechanical strength is primarily attributed to the filling of inter-particle voids by cemented calcium carbonate, which effectively constrains particle displacement and deformation, thereby inhibiting relative slippage between soil particles [44]. In contrast, the contribution to strength from the cemented calcium carbonate layer covering the soil particle surfaces is relatively minor.

#### 3.3.2. Micro-pore variations under different cementation sequences.

To quantitatively analyse the microstructural characteristics of modified loess, SEM images were processed and analysed using Image-Pro Plus 6.0 image analysis software. The specific procedure is as follows:

(1) Image pre-processing: Representative SEM images (magnification 2500×) were selected and subjected to cropping, contrast enhancement and noise reduction to eliminate interference from the image acquisition process.
(2) Binarisation: An automatic threshold segmentation method was employed to convert the images into black-and-white binary images, thereby objectively distinguishing pores from soil particles, calcium carbonate crystals and other solid-phase materials.
(3) Using the software’s analytical functions, the following microstructural parameters were extracted:
  Pore area fraction (%): The percentage of the total image area occupied by pores (Note: Analysis based on two-dimensional SEM images yields an area fraction, rather than volumetric porosity)
  Pore diameter (μm): The equivalent circular diameter of the pores
(4) Blinded observer note: During the image analysis process, the analysts were aware of the sample grouping information (i.e., the study was not blinded). This may, to some extent, introduce subjective bias and constitutes one of the limitations of this study.

Based on the above analysis, the pore area fraction (Table 5) and pore size distribution (Table 6) were calculated. Pores in loess are typically classified into four categories: micropores (< 2 μm), small pores (2–8 μm), medium pores (8–32 μm) and large pores (> 32 μm) [45].

**Table 5:** Statistical table of the percentage of pore area in specimens with different cementation sequences.

Sample type	Total pore area fraction (%)	Micropore area fraction (%)	Small pore area fraction (%)	Mesopore area fraction (%)	Macropore area fraction (%)
Control group	33.11	7.58	10.29	15.24	0
Cementation solution first, then bacterial suspension	30.35	7.12	12.52	10.71	0
Bacterial suspension first, then cementation solution	25.78	6.97	10.42	8.39	0
Simultaneous application	22.32	4.72	3.41	14.19	0

**Table 6:** Statistical table of pore counts for specimens with different cementation sequences.

Sample category	Total number of pores (units)	Number of micropores (units)	Number of small pores (units)	Number of medium pores (units)	Number of large pores (units)
Control group	4010	3996	11	3	0
Cementation solution first, then bacterial suspension	3873	3863	9	1	0
Bacterial suspension first, then cementation solution	3534	3518	14	2	0
Simultaneous application	10129	10109	15	5	0

A comprehensive analysis of the data in Tables 5 and 6 reveals that different treatment sequences in microbial-induced calcium carbonate precipitation (MICP) have a significant impact on the pore structure of Ili loess.

In terms of pore area and overall compactness, all MICP treatments effectively reduced the total pore area fraction of the soil, indicating that the MICP technique has a favourable improvement effect. Among these, the simultaneous application treatment yielded the best results, significantly reducing the pore area fraction from 33.11% in the control group to 22.32%. This is likely because this treatment sequence forms a widely distributed and uniform cementation network, achieving optimal overall filling and sealing.

Among the stepwise treatments, the bacterial suspension followed by cementation solution approach (25.78%) yielded better results than the cementation solution followed by bacterial suspension approach (30.35%). This suggests that the biofilm formed by the pre-colonisation of bacteria provided more effective nucleation sites for subsequent calcium carbonate precipitation, thereby enhancing the filling efficiency.

In terms of the evolution of pore count and structure, the treatment method induced a distinct pore size restructuring effect. On the one hand, MICP cementation primarily targets mesopores, the number and area of which generally decrease after treatment; this is a direct result of calcium carbonate precipitation filling the pores. On the other hand, the bacterial suspension followed by cementation solution treatment promotes a relative increase in the number and area of micropores; this may be due to uniformly distributed microcrystals filling the macropores whilst simultaneously refining the original pore structure.

It is worth noting that the simultaneous application treatment produced an exceptionally high number of micropores (10,129), yet their total area fraction was the lowest. This indicates that this cementation method generated smaller calcium carbonate crystals that did not coalesce into plates with other calcium carbonate precipitates; however, they exhibited higher density and more uniform distribution. Consequently, although a large number of new micropores were created, they efficiently occupied the pore space, thereby achieving optimal densification at the microscopic scale.

Changes in the pore structure directly influence changes in the macroscopic mechanical properties of the specimens. Combining the test results for the angle of internal friction and cohesion in Table 4, the angle of internal friction of the simultaneously treated specimens increased by 0.92% compared to the control group, whilst cohesion improved by approximately 55.06%. The microstructural mechanism is as follows:

(1) Although the simultaneous application treatment introduced a large number of micropores (10,129), it exhibited the lowest total pore area fraction (22.32%), indicating that these micropores were occupied by fine, densely packed calcium carbonate crystals, forming a high-density cementation network.
(2) The bacterial suspension followed by cementation solution treatment, through the nucleation effect of the biofilm, caused the calcium carbonate precipitation to concentrate more within the medium and small pores between particles, effectively enhancing inter-particle interlocking and friction.
(3) In contrast, the cementation solution followed by bacterial suspension treatment, due to the lack of bacterial pre-positioning, resulted in calcium carbonate precipitation being distributed predominantly on the particle surfaces rather than within the critical force-transfer pathways between particles; consequently, the increase in macroscopic strength was limited.

## 4. Discussion

### 4.1. Mechanism of MICP technology enhancement

Through systematic macro- and micro-scale experiments, this study confirms that the Microbial Induced Calcium Carbonate Precipitation (MICP) technique significantly improves the mechanical strength of Ili loess, consistent with existing research findings [46]. The core mechanism involves urease produced by Sporosarcina pasteurii metabolising urea to generate NH_4_⁺ and CO_3_^2-^. The latter reacts with environmental Ca^2+^ to form calcium carbonate precipitates [47], as depicted in Equations (5) and (6):


$$\begin{array}{c} CO{\left(NH_{\text{2}}\right)}_{\text{2}}+{\text{2H}}_{\text{2}}O\overset{\text{bacteria}}{\to }C\overset{\text{2}-}{\underset{\text{3}}{O}}+\text{2}NH^{\text{4}+} \end{array}$$ (5)

$$\begin{array}{c} C{\text{a}}^{\text{2}+}+C\overset{\text{2-}}{\underset{\text{3}}{O}}\to C\text{a}CO_{\text{3}} \end{array}$$ (6)

The intensification mechanism of this biomineralisation process hinges critically on the distribution pattern and crystalline morphology of calcium carbonate within the soil matrix. As indicated by Hamdan et al.’s investigation into the mechanism of enzyme-induced carbonate precipitation (EICP), the ‘cementation bridges’ formed by calcium carbonate at soil particle contact points significantly enhance inter-particle cohesion, thereby increasing soil strength [40].

### 4.2. Mechanism of cementation sequence on the improvement of Ili Loess

This study found that the cementation sequence is a key factor influencing the distribution and morphology of calcium carbonate precipitates, and consequently determines the macroscopic improvement effect of MICP. The macroscopic mechanical properties under the three cementation sequences were ranked as follows: simultaneous mixing > bacterial suspension followed by cementation solution > cementation solution followed by bacterial suspension. The fundamental reason for this pattern lies in the differential regulation of microbial reaction kinetics and mass transfer processes by the cementation sequence.

This mixing method enables the formation of a more uniform and continuous calcium carbonate network, primarily due to the synergistic interaction of the following three factors:

(1) Spatio-temporal synchronisation of reactants: Urease (produced by bacteria) and the reaction substrates (urea and Ca^2+^) are premixed prior to entering the soil, ensuring their synchronous distribution within the pore space. This avoids concentration gradients or local supersaturation caused by sequential introduction, allowing calcium carbonate precipitation to nucleate and grow uniformly throughout the soil matrix.
(2) Uniform distribution of nucleation sites: The bacteria and substrate exist as a homogeneous suspension; upon mixing with loess, the bacteria are uniformly ‘trapped’ within the pore network between soil particles. As urea and Ca^2+^ are already present in the liquid phase, once the bacteria have colonised, the conditions for local precipitation are immediately met. This ‘in situ nucleation’ mechanism ensures the microscopic uniformity of nucleation sites, thereby avoiding preferential precipitation caused by restricted substrate diffusion.
(3) Continuity and compactness of the cementation network: With uniform nucleation and synchronous reactions, calcium carbonate crystals grow in adjacent pores and interconnect, forming a spatially continuous network structure. SEM analysis reveals that in the simultaneous mixing group, calcium carbonate crystals are predominantly densely packed with a blocky or granular morphology, cementing into large lamellar structures that effectively fill the pores and bind adjacent particles. In contrast, the sequential addition method tends to result in localised ‘island-like’ precipitation, with poorer network continuity.

The method of applying the bacterial suspension before the cementation solution relies on the pre-colonisation of bacteria on the surface of soil particles, providing a wider range of nucleation sites. However, as the subsequent addition of the cementation solution involves a diffusion process, the precipitation exhibits a progressive ‘outside-in’ pattern, which affects uniformity to some extent. In the method where the cementation solution is added before the bacterial suspension, calcium ions may adsorb prematurely onto the soil particle surfaces or undergo local precipitation with soil constituents, forming a ‘coating’ on the particle surface that hinders effective contact between the bacterial suspension and the soil particles. At the same time, in the absence of sufficient bacterial catalysis, the cementation solution may partially leach away, making it difficult for the subsequent bacterial suspension to effectively utilise the pre-established environment, ultimately resulting in a loose and discontinuous cemented structure.

The calcium carbonate crystals formed during the MICP process exhibit diverse morphologies, primarily comprising calcite, aragonite and vaterite, with calcite and aragonite being the most common [48]. SEM observations in this study revealed that, in the optimal simultaneous mixing group, calcium carbonate crystals were predominantly densely packed in blocky or granular forms. They adhered to one another to form large lamellar structures, filling the intergranular voids and cementing adjacent soil particles, thereby providing effective filling and cementation within the pores and on the surfaces of the soil particles [42]. These differences in crystal morphology and distribution patterns are key microscopic factors leading to variations in the effectiveness of soil improvement across different cementation sequences. However, the dominant factors influencing crystal morphology (such as calcite and aragonite) remain unclear [36], representing an important direction for future mechanistic research.

It is worth noting that the Ili loess used in this study has a relatively high soluble salt content, which adds a unique dimension to its improvement. In untreated soil samples, easily soluble salts (such as sodium salts) can form a thick diffusion double layer on the surface of soil particles, thereby weakening the interparticle molecular forces [49].

The MICP amendment process not only generates calcium carbonate cement, but the Ca^2+^ introduced may also displace Na⁺ from the surfaces of soil particles. It is hypothesised that the reduction in sodium ion content helps to compress the double layer and reduce the spacing between soil particles, thereby enhancing interparticle interactions [50]. Consequently, the improvement of Ili loess by MICP may be a process involving both physical and chemical mechanisms: on the one hand, the physical filling and cementation of calcium carbonate precipitates; on the other hand, the ion exchange of low-valent sodium ions by high-valent calcium ions and the chemical optimisation of the soil structure. These two mechanisms work synergistically to improve the pore structure and comprehensively enhance the mechanical strength of the soil samples [51].

It should be noted that the above analysis of the mechanisms underlying ion exchange and double-layer compression is currently based primarily on theoretical deductions and comparisons with the literature [49–51], and is not yet supported by direct chemical measurement data on the concentrations of Na⁺ and Ca^2+^ or the cation exchange capacity (CEC) in the soil samples before and after treatment in this study. Consequently, this explanation remains a reasonable conjecture based on existing observations, and the relevant conclusions require further verification in subsequent studies (see Section 4.4 for details).

### 4.3. Recommendations for engineering applications

#### 4.3.1. The applicability of the spray-mixing method.

The loess in Ili has fine pores and low permeability, making it difficult for traditional grouting methods to ensure the uniform migration of bacterial suspensions and cementation solutions [52]. Consequently, this study employed the spray-mixing method, whereby the reactants were uniformly sprayed and mixed into the soil sample prior to compaction, thereby fundamentally circumventing the constraints imposed by low permeability on solution migration. This method achieved the dual objectives of ‘mechanically optimal compaction’ (18.77% optimal moisture content) and ‘chemically uniform cementation’. Although it differs from on-site grouting processes, the research findings are applicable for translation into practical engineering applications.

#### 4.3.2 Engineering Implementation of the ‘Simultaneous Mixing Method’.

Laboratory tests have shown that the ‘simultaneous mixing method’ (where the bacterial suspension and cementation solution are pre-mixed before being blended with the soil) is the optimal process. The following recommendations are made for practical engineering applications:

(1) Equipment: Portable mixers may be used for small-scale projects; for large-scale projects, it is recommended to use a mobile dual-fluid mixing plant to achieve continuous and uniform mixing via a static mixer.
(2) Proportions: Maintain a 1:1 mass ratio of bacterial suspension to cementation solution (OD_600_ = 1.1, 0.5 M urea + 0.5 M CaCl_2_), controlled by a flow meter.
(3) Procedure: Solution preparation → mixing and stirring for at least 30 seconds → uniform spraying and mixing for 30 minutes → spreading and compaction → curing under plastic sheeting for at least 7 days.
(4) Quality control: Regularly test the pH of the mixed solution, the moisture content of the mixed soil sample (target value ±1%), and the strength after 7 days of curing.

It is recommended that on-site pilot trials be conducted prior to full-scale construction to validate and optimise the process parameters**. It should be emphasized that these recommendations are based on laboratory findings; field validation, durability assessments, and scale-up studies are essential before practical engineering application.**

### 4.4. Limitations of the study and future prospects

Although this study has systematically revealed the influence of cementation sequence on the mechanical properties of MICP-amended Ili loess, further research is required in several areas:

In terms of experimental design, this study did not include an abiotic/sterile control group (such as treatments using a cementation solution without live bacteria, or treatments with heat-inactivated bacteria). Consequently, it is not possible to quantitatively distinguish the respective contributions of true biomineralisation effects, chemical precipitation effects, or physical compaction effects to the increase in strength. Previous studies have shown that treatment with live bacteria using the MICP method produces significantly higher calcium carbonate precipitation and improved mechanical properties compared to sterile control groups; however, under the specific soil conditions of the Ili Loess, direct comparative data for these effects are still lacking. It is recommended that future studies incorporate such control groups to further verify the exclusive role of microbial activity in the consolidation of loess in this region.

In terms of mechanistic analysis, this study hypothesises that the compression of the double electric layer through the replacement of Na⁺ by Ca^2+^ is one of the auxiliary strengthening mechanisms of MICP treatment; however, it lacks chemical analysis data on the soluble salt content (Na⁺ and Ca^2+^ concentrations) and cation exchange capacity (CEC) of soil samples before and after treatment. It is recommended that subsequent studies systematically measure the aforementioned chemical indicators before and after MICP treatment to quantitatively assess the extent to which ion exchange contributes to the consolidation effect, thereby providing a more comprehensive understanding of the microscopic mechanisms underlying the MICP technique in loess consolidation.

At the microstructural characterisation level, this study employed SEM to examine the morphology of calcium carbonate only, without conducting quantitative analyses such as XRD or thermogravimetric analysis (TGA). Consequently, further research is required to investigate the phase composition of calcium carbonate polymorphs (calcite, aragonite, and vaterite) and their quantitative relationship with mechanical properties. It is recommended that future studies combine XRD phase analysis with quantitative testing of calcium carbonate using acid digestion and TGA to provide a more comprehensive understanding of the microstructural mechanisms underlying MICP-treated loess.

With regard to environmental durability, it is necessary to assess the impact of the coupled ‘wet-dry and freeze-thaw cycles’—a unique environmental phenomenon in the Ili River Valley—on the long-term durability of the cemented structure. Environmental coupled accelerated ageing tests should be conducted, and combined with microscopic characterisation techniques to reveal the evolution patterns and failure modes of the calcium carbonate cementation network under repeated environmental stresses.

In terms of process optimisation, it is necessary to conduct in-depth investigations into issues such as microbial colonisation patterns, mass transfer of reactants and crystal growth kinetics under different cementation sequences, thereby elucidating the microscopic mechanisms underlying macroscopic phenomena. Concurrently, a synergistic optimisation model must be established that integrates multiple parameters—including cementation sequence, bacterial suspension activity, cementation solution concentration and soil condition—to develop a multi-objective optimisation framework using response surface methodology or machine learning, thereby formulating a detailed process guideline.

At the engineering application level, field trials should be conducted to verify the technical feasibility, uniformity of reinforcement and long-term effectiveness of different cementation sequences in practical engineering projects. This will facilitate the engineering application of this technology in the Ili Loess Plateau region and provide reliable technical support for soil and water conservation and the prevention and control of geological hazards in the arid and semi-arid areas of Northwest China.

## 5. Conclusion

This study systematically investigated the effect of cementation sequence on the efficacy of Microbial Induced Calcium Carbonate Precipitation (MICP) technology for improving Ili loess through macro- and micro-scale experiments. The following key conclusions were drawn:

(1) The cementation sequence significantly influenced the MICP improvement effect. The macro-mechanical properties of the three cementation sequences ranked as follows: simultaneous mixing > bacterial suspension followed by cementation solution > cementation solution followed by bacterial suspension. Specifically, specimens treated with simultaneous mixing achieved an unconfined compressive strength of 115.83 kPa, representing an approximately 59.77% improvement over untreated specimens. These specimens also exhibited the highest deviator stress and cohesion in triaxial shear tests.
(2) Microstructural analysis revealed the underlying mechanisms for these performance differences. SEM analysis indicates that the sequence of cementation influences the improvement effect by controlling the distribution and morphology of calcium carbonate precipitation. Simultaneous mixing formed a continuous, dense cementing network dominated by densely packed calcium carbonate crystals, achieving optimal pore filling and effective particle bridging. In contrast, sequential treatment often resulted in uneven cement distribution or reduced calcium carbonate formation, thereby limiting strength enhancement.
(3) The core of the improvement effect lies in the integrity of the cementation network. The enhancement in mechanical properties primarily depends on the formation of effective ‘cementation bridges’ between soil particles via calcium carbonate and the continuity of the spatial network they constitute, rather than solely on the total amount of calcium carbonate generated or its surface coverage. The simultaneous mixing process demonstrates the greatest advantage in constructing a complete three-dimensional cementation network.
(4) MICP technology combines cementation enhancement with environmental sustainability. Through biomineralisation, it specifically increases cohesion within the soil (maximum improvement of 55.06%) while minimally affecting internal friction angle (variation <0.92%). This demonstrates its potential as a green reinforcement method that improves soil cementation capacity while largely preserving its original friction characteristics.

## Supporting information

S1 Data Raw test data of unconfined compressive strength and triaxial shear strength for MICP-modified Ili loess. (XLSX)

S2 Data Parallel specimen test data of unconfined compressive strength and triaxial shear strength for MICP-modified Ili loess. (XLSX)
